# Reliability of *My Jump* 2 Derived from Crouching and Standing Observation Heights

**DOI:** 10.3390/ijerph19169854

**Published:** 2022-08-10

**Authors:** Jose M. Jimenez-Olmedo, Basilio Pueo, Jose M. Mossi, Lamberto Villalon-Gasch

**Affiliations:** 1 Physical Education and Sport, University of Alicante, 03690 Alicante, Spain; 2 ITeam, Institute of Telecommunications and Multimedia Applications, Universitat Politècnica de València, 46022 Valencia, Spain

**Keywords:** smartphone, jump, error, test, within-observer, between-observer, measurement

## Abstract

The crouching or prone-on-the-ground observation heights suggested by the *My Jump* app are not practical in some settings, so users usually hold smartphones in a standing posture. This study aimed to analyze the reliability of *My Jump* 2 from the standardized and standing positions. Two identical smartphones recorded 195 countermovement jump executions from 39 active adult athletes at heights 30 and 90 cm, which were randomly assessed by three experienced observers. The between-observer reliability was high for both observation heights separately (ICC~0.99; SEM~0.6 cm; CV~1.3%) with low systematic (0.1 cm) and random (±1.7 cm) errors. The within-observer reliability for the three observers comparing the standardized and standing positions was high (ICC~0.99; SEM~0.7 cm; CV~1.4%), showing errors of 0.3 ± 1.9 cm. Observer 2 was the least accurate out of the three, although reliability remained similar to the levels of agreement found in the literature. The reliability of the mean observations in each height also revealed high reliability (ICC = 0.993; SEM = 0.51 cm; CV = 1.05%, error 0.32 ± 1.4 cm). Therefore, the reliability in the standing position did not change with respect to the standardized position, so it can be regarded as an alternative method to using *My Jump* 2 with practical added benefits.

## 1. Introduction

The accurate measurement of human movement is an essential part of sport and exercise science professionals to monitor, assess, and develop training programs. A number of tests have been developed to measure and evaluate physical performance and fitness, either in laboratory settings or in the field [1]. Vertical jump tests are reliable methods used in many populations, from school children [2] to the elderly [3], and athletes from various disciplines [4,5]. Vertical jump performance has been used to identify talent in sports such as football [6], evaluate lower limb power [7], and monitor neuromuscular fatigue to avoid injuries in athletes [8]. Considering the simplicity of vertical jump tests, coaches and strength and conditioning professionals use this measure as a key part of any performance analysis.

The tracking of the body’s center of mass through infrared videocameras and the integration of the ground reaction force measured on a force plate are the most accurate methods to measure vertical jump height [7]. The limited access to these laboratory methods, the huge cost of instruments, or the need for expert personnel to operate them has led to the development of lower-cost alternatives, such as jump mats [9], photocells [10], accelerometers [11], or linear position transducers [12]. The first two methods rely on the detection of jump flight time by identifying the take-off and landing instants in standardized jump executions, such as squat jump (SJ) or countermovement jump (CMJ). With the advent of smartphones and tablets able to capture high-speed video recordings at high image resolution, the flight time of a jump execution could be manually assessed through the identification of video frames at take-off and landing. Under this premise, *My Jump* was released as a portable, easy-to-use, low-cost alternative smartphone app for iOS (Apple Inc., Cupertino, CA, USA) for practitioners wanting to monitor jump height in all kinds of fields [13]. *My Jump* has not only been tested in adult athletes [4,5], but also in other populations, such as school children [2], trained junior athletes [14], the elderly [3], and cerebral palsy players [15].

The performance of *My Jump* has been validated in the literature against laboratory gold-standard criteria [13,16,17], deriving low systematic bias and technical errors around 1 cm [18]. Regarding reliability, the app has shown high intra-session reliability (intraclass correlation coefficient ICC close to unity and low coefficient of variation CV) tested either by two independent observers: ICC = 0.995, CV = 3.4–3.6% [13] or by the same observer: ICC = 0.97, CV = 3.9% [19]. Similarly, the inter-session reliability was also high for sessions separated by 48 h: ICC = 0.99, CV = 6.7% [20] and ICC = 0.97–0.99, CV = 3.8–7.6% [16], or 7 days: ICC = 0.996, CV 4.9–4.5% [14], ICC = 0.99 [17]. On all occasions, following the guidelines of *My Jump* [13], researchers lay prone on the ground or crouch while holding the smartphone or simulating that position with tripods at around 30 cm from the ground.

However, the prone-on-the-ground or crouching positions are not practical in some settings, particularly for massive measurements, so practitioners usually hold the smartphone in a standing posture, trying to lower the height to the minimum possible height that allows the visualization of the screen. Although the recorded point of view may look similar to that close to the ground, it remains unclear if the high reliability of the app for crouching position still holds for this elevated point of view.

To the authors’ knowledge, no other studies have assessed the reliability of *My Jump* in a standing position. Therefore, the aim of this study was to analyze the reliability of *My Jump* 2 for active adult athletes from the standardized and standing positions with three independent observers. We hypothesized that *My Jump* 2 would show high reliability when used in a standing position for CMJ heights.

## 2. Materials and Methods

### 2.1. Participants

Thirty-nine active athletes in various disciplines participated in this study, distributed as 25 male athletes (age 22.2 ± 2.7 y, body mass 77.6 ± 6.8 kg, height 180.1 ± 4.4 cm), and 14 female athletes (age 23.2 ± 1.8 y, body mass 66.2 ± 4.0 kg, height 170.7 ± 4.4 cm). The inclusion criteria were as follows: noncompetitive athletes participating in recreational resistance training and aerobic exercise (running, rowing, cycling and team sports such as soccer and basketball), no lower extremity injury for the past 6 months, and lack of lower and upper limb pain. All jumps were executed by each participant at the same time of the day to avoid any effect of circadian variation and in similar ambient conditions of temperature (~22 °C) and relative humidity (55–60%). Subjects were told to refrain from drinking alcohol or caffeinated beverages for 24 h before the testing session. The study was carried out in accordance with the guidelines of the ethical principles of the Declaration of Helsinki (2000). All subjects provided informed consent before the beginning of this study, which was approved by the University Institutional Review Board (IRB No.UA-2019-02-25).

### 2.2. Study Design

This was an observational study to assess the reliability of observations using *My Jump* 2 at two smartphone heights, one squatting, following the manufacturer guidelines, and another standing up. Participants attended the laboratory in a single test session to execute CMJ. High-speed videos were recorded by two identical smartphones at the two observation heights. Three independent trained observers assessed each recording using the app running on each smartphone.

### 2.3. Instrumentation

**Figure 1:**
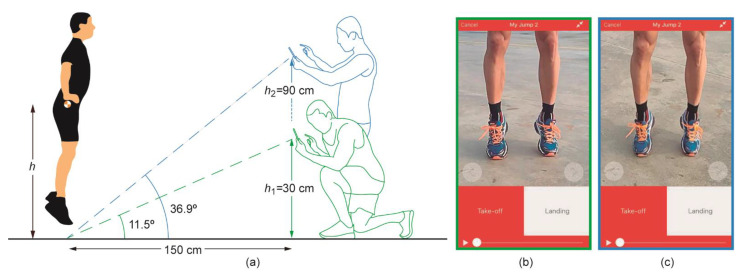
(**a**) Graphical illustration of the experimental setup for observations at two observation heights and their associated screenshots of the same jump execution for *h*_1_ (**b**) and *h*_2_ (**c**). Heights and angles are not to scale. Both smartphones were located on tripods at the two observation heights.

Two *My Jump* 2 apps running on two iPhone 7 units (Apple Inc., Cupertino, CA, USA) were used to record and process high-speed videos (240 fps, 720p) of jump executions [18]. The first smartphone was located on a tripod at 30 cm height (*h*_1_) following the guidelines of the app [13], resulting in an angle of 11.5 degrees from the ground, as shown in Figure 1a. The second smartphone was located on another tripod at 90 cm in height (*h*_2_) allowing the observer to record videos while standing. This height increases the observation angle to 36.9 degrees. The smartphone screenshots at the two observation heights are depicted in Figure 1b for *h*_1_ and Figure 1c for *h*_2_.

### 2.4. Methodology

A standardized 10 min warm-up composed of resistance exercises followed by a cycle ergometer set up at 80 W load, joint mobilization exercises, and several familiarization jumps was executed [21]. Then, participants performed five CMJ with a rest period of 1 min between trials. The jump protocol included real-time video analysis in the sagittal plane to ensure participants flexed their knees to 90 degrees, and afterward, they jumped to maximum effort in a continuous movement with hands akimbo. Participants were instructed to repeat any jumps that did not follow the above guidelines.

The recordings were assessed on the same smartphone of the recording phase by three experienced observers in the use of *My Jump*. Before the assessment, observers agreed on the criteria commonly used to select video keyframes to ensure consistency in the observations. The first frame displaying both feet off the ground was selected as the take-off instant and at least one foot touching the ground as the landing instant [22]. Each of the 3 observers assessed each of the 195 jumps (5 jumps per 39 participants) recorded simultaneously at heights *h*_1_ and *h*_2_, so the number of observations was 1170. All observations were conducted independently to avoid mutual influences. The videos were randomly analyzed in regard to the observation heights, jump trials, and participants.

### 2.5. Statistical Analysis

The reliability of observations with *My Jump* 2 under different conditions was assessed through a set of statistics testing the level of agreement and the magnitude of errors [23]. With regards to the agreement, correlation analysis includes Pearson’s (*r*), intraclass (ICC), and concordance (CCC) correlation coefficients. Bivariate Pearson’s product-moment correlation coefficient *r* and linear regression analysis were used to study the linear relationship between paired observations. Ordinary Least Squares regression was used to estimate coefficients *β*_0_ (intercept) and *β*_1_ (slope) of predictive linear regression equations *y* = *β*_0_ + *β*_1_*x*. The standard deviation of the residuals was calculated as the standard error of estimate (SEE) to assess how well a linear regression model fits the data. Intraclass correlation coefficient ICC (2,*k*), 2-way random-effects, and absolute agreement were used since the observers of this study were part of a larger population of observers with similar characteristics using *My Jump* 2 [24]. In this model, *k* multiple raters was only used for mean observations across observers, whereas for single observers, the model was ICC (2,1). Lin’s concordance correlation coefficient CCC was calculated to assess the agreement as a function of the means, variances, and covariances of the bivariate distribution of two observations [25]. For practical purposes, CCC evaluates how closely related two variables are in a linear fashion and the degree to which pairs of observations fall on the 45° line through the origin. The following thresholds were used in the correlation analysis for the assessment of technological equipment in research and clinical practice: very poor <0.70, poor 0.70–0.90, moderate 0.90–0.95, good 0.95–0.99, and very good >0.99 [26].

Regarding the magnitude of errors, absolute reliability was assessed with the standard error of measurement (SEM), computed as the standard deviation of the paired differences divided by √2 [27]. SEM is a measure of how much repeated measures are spread around the true value [28]. The standardized version of SEM was also computed, whose threshold for trivial disagreement was <0.2 [27]. SEM is also expressed as a percentage of the mean or coefficient of variation CV (SEM/mean). For many measurements in sports medicine and science, high reliability was determined as ICC > 0.90 and CV < 5% [27]. Sensitivity was calculated using two indicators, the smallest detectable change (SDC) and the smallest worthwhile change (SWC). SCD was calculated as 1.96 × √2 × SEM [29], representing the minimal change in jump height that an athlete must perform to ensure that the observed change is real and not just a measurement error [30]. Similarly, SWC was calculated as 0.2 of the between-subjects standard deviation [28,31], representing the minimum improvement likely to have a practical impact [32]. The usefulness was assessed by comparing the sensitivity (SWC) and its associated noise (SEM): if sensitivity was greater than noise, the ability of the method to detect small performance changes in jump height was rated as good [33,34]. Paired samples *t*-test and standardized mean differences (Hedges’ *g* corrected effect size [35]) were used to compare observations. The effect size (ES) was interpreted as trivial if *g* < 0.2 [36]. The agreement between observations was also studied with Bland–Altman plots through analysis of differences of observation pairs against their mean values [29,37], identifying random errors, and proportional bias between observations if bivariate Pearson’s product-moment correlation coefficient was *r*^2^ > 0.1 [28,37].

Both the level of agreement and the magnitude of errors were evaluated for the following situations. The between-observer reliability was assessed by comparing the observations arising from paired observers 1 vs. 2, 1 vs. 3, and 2 vs. 3 for the two observation heights independently. Once the consistency of the outcomes generated by each observer was verified, the within-observer reliability for the two methods (observation heights) was assessed by comparing the observations from *h*_1_ vs. *h*_2_ for the three observers independently. Finally, the method reliability was evaluated by comparing the mean of observations <*h*> from the three observers between the two observation heights.

The Shapiro–Wilk normality test was used, which resulted in a normal distribution. All statistical analyses were computed with an available spreadsheet for reliability [38] and with SPSS v. 22 (IBM Corp., Armonk, NY, USA).

## 3. Results

### 3.1. Between-Observer Reliability

Table 1 shows the results for between-observer reliability for observation heights *h*_1_ and *h*_2_ separately. The differences between observers were very low, being 0.16 cm between observers 1 and 3 for *h*_2_ the maximum value, all assessed as trivial (ES ranging from 0.01 to 0.03). ICC and CCC showed good (0.980 to 0.988) to very good (0.992 to 0.994) agreement for all observers. SEM was below 0.46 to 0.86 cm, all assessed as trivial disagreement in the standardized version (<0.2). Additionally, CV ranged from 0.76% to 1.58%, meaning high reliability for both observation heights. Sensitivity via SDC (1.27 to 2.03 cm) and SWC (around 1.21 cm) indicated the amount of jump height that *My Jump* 2 can detect over the noise. Thus, the resulting SNR was greater than unity: 1.40 to 2.62.

**Figure 2:**
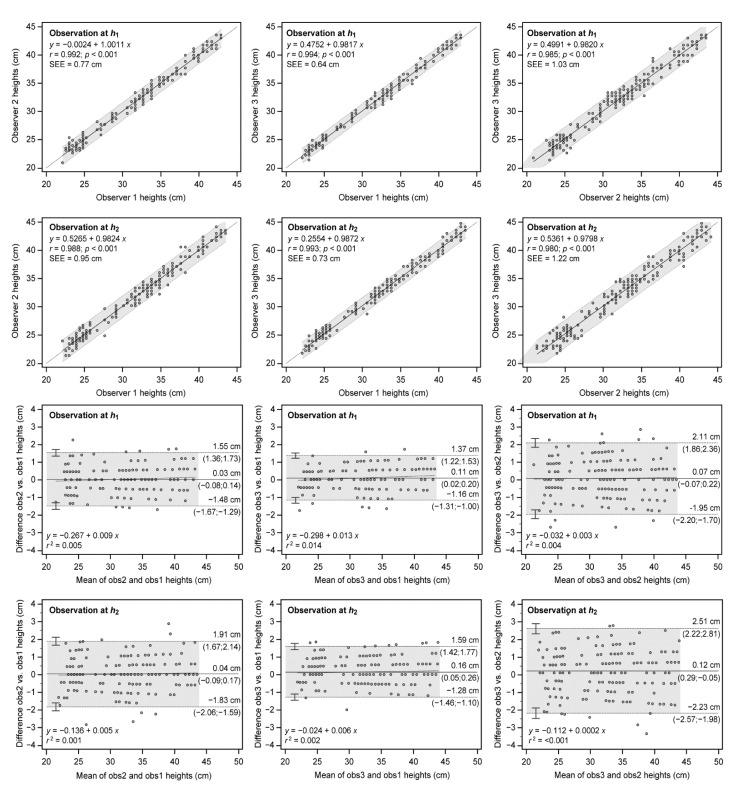
Agreement between observers derived from the two observation heights *h*_1_ and *h*_2_. Relationship between observations (**top**) and Bland–Altman plots (**down**) are shown. For regression, the dotted line represents linear regression; upper and lower dashed lines show 95% confidence intervals. Predictive linear regression equations, Pearson’s product-moment correlation coefficient (*r*) and standard error of estimate (SEE) are also depicted. For Bland–Altman, the solid central line represents the mean between observations (systematic bias); upper and lower dashed lines show mean ± 1.96 SD (random error) with CI-95% limits in parentheses; the dotted line depicts linear regression (proportional bias). Linear regression equations of the differences and determination coefficient *r*^2^ are also shown to inspect bias proportionality.

The results of between-observer agreement in regard to scattered plots of paired observations from the two heights and associated Bland–Altman plots are depicted in Figure 2. Results showed a good and very good linear relationship between paired observations in both observation heights (0.980 to 0.994) with intercepts around 0.5 cm and slopes close to unity. The standard deviation of the residuals remained low (0.64 to 1.22 cm) so the linear regression model was able to fit the data. Bland–Altman plots also showed high agreement between observations provided most of the paired observations remained inside the ±95% limits of agreement. In addition, low systematic bias (−0.18 to 0.03 cm), random errors (±1.2 to ±2.4 cm), and lack of proportional bias (*r*^2^ < 0.1) were observed.

### 3.2. Within-Observer Reliability

The agreement between the two observation heights (methods) for each observer is shown in Table 1. The paired differences of observations arising from the two heights were larger than those of between-observer for *h*_1_ and *h*_2_ separately, but still low in magnitude (0.31 to 0.35 cm) and trivial (ES 0.05 to 0.06). Likewise, ICC and CCC were good for observers 2 and 3 (0.977 to 0.983) and very good for observer 1 (0.993 to 0.995). In spite of such differences found in observers, reliability was high. Similarly, SEM was lower for observer 1 (0.44 cm) than for the rest of the observers (0.81 and 0.89 cm), although all coefficients can be assessed as trivial (ES 0.07 to 0.15). The percentage SEM of the mean or CV was also similar to the between-observer reliability (0.85 to 1.69%). Finally, SDC and SWC were low in general (1.20 to 2.45 cm), resulting in an SNR ranging from 1.33 for observer 2 to 2.76 for observer 1, yet the signal was greater than noise for all observers.

**Figure 3:**
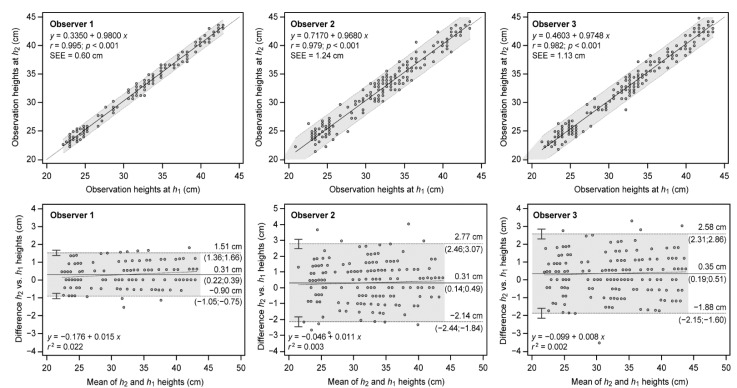
Agreement between methods derived from observers 1, 2, and 3. Relationship between observations (**top**) and Bland–Altman plots (**down**) are shown. For regression, dotted line represents linear regression; upper and lower dashed lines show 95% confidence intervals. Predictive linear regression equations, Pearson’s product-moment correlation coefficient (*r*) and standard error of estimate (SEE) are also depicted. For Bland–Altman, solid central line represents the mean between observations (systematic bias); upper and lower dashed lines show mean ± 1.96 SD (random error) with CI-95% limits in parenthesis; dotted line depicts linear regression (proportional bias). Linear regression equations of the differences and determination coefficient *r*^2^ are also shown to inspect bias proportionality.

The scattered plots depicted in Figure 3 indicated a good to very good linear relationship between heights for observers 2 and 3 (0.979 and 0.982), and observer 1 (0.995), respectively. In all cases, intercepts were around 0.5 cm (0.33 to 0.71 cm) and slopes near unity (0.968 to 0.980). As with the between-observer reliability, the linear regression model fit the data to a large extent (SEE from 0.60 to 1.24 cm). Likewise, Bland–Altman plots showed high agreement for the three observers, limits of agreement being narrower for observer 1 (±1.20 cm) than for the rest (±2.45 and ±2.23 cm), although the three observers demonstrated high reliability. Finally, systematic bias was low (0.31 to 0.35 cm) and the absence of association between the magnitudes of errors was found (*r*^2^ from 0.002 to 0.022).

### 3.3. Method Reliability

**Figure 4:**
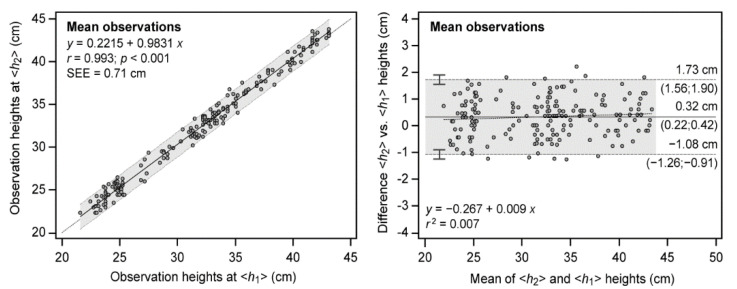
Method agreement between mean observations derived from the two observation heights *h*_1_ and *h*_2_. Relationship between observations (**left**) and Bland-Altman plots (**right**) are shown. For regression, dotted line represents linear regression; upper and lower dashed lines show 95% confidence intervals. Predictive linear regression equations, Pearson’s product-moment correlation coefficient (*r*) and standard error of estimate (SEE) are also depicted. For Bland–Altman, solid central line represents the mean between observations (systematic bias); upper and lower dashed lines show mean ± 1.96 SD (random error) with CI-95% limits in parentheses; dotted line depicts linear regression (proportional bias). Linear regression equations of the differences and determination coefficient *r*^2^ are also shown to inspect bias proportionality.

Since the observers showed high within- and between-observer reliability, the following showed the agreement between the two heights or methods for the mean of the three observations per height. Table 1 shows that paired differences were 0.32 cm (CI-95% 0.22 to 0.42 cm, *p* < 0.05), as the bias between methods which can also be observed in the Bland–Altman plot of Figure 4. The disagreement is quantified as trivial, according to ES of 0.05. Very good agreement (>0.99) [26] was observed for both ICC (0.993) and CCC (0.992). Likewise, the precision of the observations was high since SEM resulted in 0.51 cm, quantified as trivial (*g* < 0.2), or alternatively, expressed as a percentage of the mean CV, which was around 1%. The measurement error was also assessed through SDC and SWC. Results indicated that the variation expected when using *My Jump* 2 under the two conditions due to measurement error was 1.41 cm. Similarly, the minimum practically meaningful change in jump height due to personal enhancements over the noise of the observation was SWC = 1.20 cm. Therefore, *My Jump* 2 was able to detect changes over the standard error of measurement (noise) since SNR = 2.37.

The scattered plot shown in Figure 4 indicated a very high association between observations at the two heights (0.993, *p* < 0.001). Likewise, the predicting linear regression equation was accurate since the slope was near unity, the intercept was also close to the method bias, and SEE was 0.71 cm. Bland–Altman plots showed a high level of agreement between methods since most of the paired observations fell inside the 95% limits of agreement, depicted in the gray shaded area. Similarly, a low mean systematic bias of 0.32 cm and random errors of ±1.4 cm were observed. The difference in observations between the two methods remained constant with increasing jump height (*r*^2^ = 0.007) because of the homoscedasticity of the errors.

## 4. Discussion

The aim of this study was to assess the reliability of *My Jump* 2 from the standardized observation height according to the app guidelines (*h*_1_) and a more practical elevated standing observation height (*h*_2_). From the analysis of outcomes from both observation heights simultaneously by three independent observers, the reliability of *My Jump* 2 could be extended to a new observation scheme *h*_2_. The major finding of this study is that the levels of agreement and the magnitude of errors for the standing position remained similar to those obtained in the standardized observation height, fulfilling the study hypothesis.

Some reliability studies with *My Jump* have used Koo and Li’s guidelines for interpretation of ICC values: <0.50, 0.50–0.75, 0.75–0.90, and >0.90 representing poor, moderate, good, and excellent ICC, respectively [14,19,24,39,40]. In our study, we adopted higher ICC values to assess technological equipment in research and clinical practice: very poor <0.70, poor 0.70–0.90, moderate 0.90–0.95, good 0.95–0.99, and very good >0.99 [26], similarly to reliability studies of other sports science studies [23,41]. For this reason, comparisons with published studies are focused on ICC value, rather than the associated qualitative assessment.

We used a study design with three trained observers who independently assessed each of the 195 jumps in both observation heights. In order to analyze if the sample of observers gave reliable outcomes, the between-observer reliability was first conducted for both *h*_1_ and *h*_2_. The level of agreement between the three observers was high, given the values of ICC and CCC close to unity for *h*_1_ as in the study of Balsalobre-Fernández et al. conducted with adult athletes [13], which showed 0.995. For the observation height under test, *h*_2_, ICC, and CCC ranged between 0.980 and 0.993, assessed as good and very good agreement, respectively. The bivariate Pearson’s product-moment correlation coefficients between paired outcomes were ~0.99 for observers 2 vs. 1, and 3 vs. 1, and ~0.98 for observers 3 vs. 2 in both observation heights, meaning that the observation accuracy remained stable for the two observation heights. There was also a nearly perfect fit of the linear regression equations with paired observations, showing slopes close to unity (0.9798 for observer 3 in *h*_2_ to 1.0011 for observer 1 in *h*_1_) and the standard deviation of the residuals was around 1 cm. These results are in agreement with Brooks et al. [20], who reported a slope of 0.91 for test-retest with *My Jump* 2. The magnitude of errors for both observation heights was low. SEM was consistent between *h*_1_ (0.46 to 0.73 cm) and *h*_2_ (0.52 to 0.86 cm), showing all trivial disagreements as standardized SEM was below 0.2 (0.09 to 0.14). This result is slightly lower than the study of Rago et al. [19], which showed SEM of 0.5 cm for test–retest reliability in a single session with adult athletes. Since ICC > 0.90 and CV < 5% for both observation heights, the between-observer reliability is deemed as high [27]. The three observers in both *h*_1_ and *h*_2_ were sensitive enough to monitor variations in jump height over the uncertainty of the measuring process [33] as SNR was greater than unity (1.40 to 2.62). Paired differences between observers 1 and 2 were slightly higher than for the two other comparisons involving observer 3. However, all were assessed as trivial since *g* < 0.2 [36], so all three observers are deemed interchangeable (mean differences ranging from 0.03 to 0.16 cm), in accordance with other studies conducted with two observers: 0.1 cm [13]. The observations at *h*_2_ showed a minor overestimation of jump height compared with *h*_1_ (0.16 vs. 0.11 cm in the worst-case scenario), which falls below typical test-retest paired differences between sessions: 0.2 cm [14,16] and 0.3 cm [20]. Bland–Altman was also used to assess the systematic bias and random effects derived from observations at *h*_1_ and *h*_2_. All observers showed very low systematic bias (0.03 to 0.16 cm), which is negligible in comparison to typical jump height ranges [42]. Additionally, random errors were low for all observers and observation heights, although narrower limits of agreement are found for observers 1 and 2, meaning that the reliability of those observers is slightly higher. The random error of the proposed observation height *h*_2_ was larger than the standardized height *h*_1_ (increments of ±0.36, ±0.17, and ±0.34 cm for pairs obs2 vs. 1, obs3 vs. 1, and obs3 vs. 2, respectively). This small increase is likely due to additional uncertainty in standing position. Finally, the errors for all comparisons were homoscedastic due to the lack of association between the bias and random errors (*r*^2^ < 0.1) [28]. Hence, the amount of random error for all observations is low, even for the range of jump height measurement [43].

Given that observers showed proper reliability observation heights *h*_1_ and *h*_2_ separately, we were able to compare the paired observations from *h*_1_ vs. *h*_2_ for each observer independently (within-observer reliability) and for the mean observations among observers <*h*_1_> vs. <*h*_2_> (method reliability). The level of agreement between methods was marginally higher for observer 1 than for the rest of the observers (lower paired differences and higher ICC/CCC), although all remained highly reliable (ICC ranging from 0.995 for observer 1 to 0.979 for observer 2). With regards to the method reliability, both ICC and CCC demonstrated very good reliability (>0.99) [26], in accordance with other reliability studies with *My Jump*: 0.995 [13], 0.99 [20], 0.996 [14], and 0.99 [17]. According to the bivariate Pearson’s product-moment correlation coefficient between paired outcomes from each observer (0.995, 0.979, and 0.982), and from the mean (0.993), the standing position *h*_2_ provided valid measures of jump height. Similarly, the linear regression model between observation heights revealed nearly perfect fit, as slopes were near unity (0.98, 0.97, and 0.97, for observers 1, 2, and 3, respectively) and the standard errors of estimate were low (0.60 to 1.24 cm). The analysis for mean observations provided similar results (slope 0.98, SEE 0.71 cm), together with an intercept of 0.22 cm, in agreement with a paired difference of 0.32 cm [28]. The magnitude of errors derived from the two observation heights revealed low systematic and random errors, as well as high sensitivity to make meaningful observations over the noise of the measure. The uncertainty of the observation was low, given that SEM was below centimeter for all observers (0.44, 0.89, and 0.81, for observers 1, 2, and 3). SEM for the mean observations (0.51 cm) was in accordance to other studies of test-retest with *My Jump* conducted with junior athletes: 0.5 m [14]. When SEM is expressed as a percentage of the mean, our results showed an uncertainty ranging from 0.85% to 1.69%. These findings differ with similar test–retest studies conducted in a single session either by two independent observers (3.4–3.6% [13]), or by the same observer (3.9% [19]). The disagreement may be explained by the lower frame rate of 120 fps used in these two studies, contrastingly to the present study which used 240 fps [18]. It is worth mentioning that the method reliability was high, given that ICC > 0.90 and CV < 5% for all observers and for the mean observations [25]. Our findings also showed that the smallest detectable change SDC and the smallest worthwhile change SWC ranged between 1.20 and 1.41 cm, so the minimum improvement in jump performance likely to have a practical impact was greater than with jump mats (0.56 cm [44]), as the uncertainty of manual digitization is expected to exceed that of electromechanical systems. Rago et al. reported similar values of SWC (0.8 cm) and SDC (1.5 cm) in a test–retest reliability study with *My Jump* [19]. The usefulness of the standing position *h*_2_ was addressed by comparing the uncertainty of the measure (SEM) and the minimum meaningful change in jump performance (SWC). Our findings demonstrated signal-to-noise ratios ranging from 1.33 to 2.76 for observers 2 and 1, respectively, which are in agreement with SNR found in another reliability study (0.08/0.05 = 1.6 [19]). All paired differences between the two observation points were ~0.3 cm (*p* > 0.05), assessed as trivial (*g* < 0.2). Our results are in concordance with test–retest paired differences between sessions using *My Jump* (0.2 cm [14,16], 0.3 cm [20], and 0.43 cm [17]). In addition to this low systematic bias between observation heights, the random errors given Bland–Altman plots depicted limits of agreement of ±1.2, ±2.4, and ±2.2 cm for observers 1, 2, and 3, respectively, which were similar to another reliability study: ±1.05 cm [17], but slightly higher than the first study with *My Jump*: ±0.4 cm [13], both conducted with adult athletes. There was no association between differences and means of paired observations for both heights (*r*^2^ < 0.1), hence the amount of random error did not change over the range of measurement. This feature is of the utmost importance to assess small improvements in jump height for trained athletes [43].

The reliability studies with *My Jump* have been conducted with a variety of smartphone models, ranging from the first iPhone with slow motion capabilities (iPhone 5, 4″ screen, 1136 × 640 pixel resolution [13]) to newer models (iPhone 6 and 6s, 4.7″ screen, 1334 × 750 pixel resolution [16,17,19,45]). Other studies have used iPads with larger screens: from iPad mini (7.9″ screen, 2048 × 1536 pixel resolution [39]) to iPad Pro (12.9″ screen, 2732 × 2048 pixel resolution [14,20]). The remarkable difference in display sizes and resolutions makes it difficult to derive balanced comparisons with this study. The reliability and accuracy of frame selection may be influenced by the instrument, being a smartphone or tablet, so therefore, the findings of our study should be verified for larger screens. Another study limitation is the use of countermovement jump only. While this type of jump is one of the most used by sports professionals and scientists, the squat jump or the countermovement jump with arms is often included in training programs. Finally, in order to strengthen the statistical accuracy, the number of observers with different expertise levels may be increased.

## 5. Conclusions

This study showed that a standing position to hold the smartphone while using *My Jump* is a reliable method to assess vertical jump height which shows similar levels of agreement to those of standardized, prone-on-the-ground position.

Minor differences were found between any of the three experienced observers in both levels of agreement and magnitude of errors. The reliability of *My Jump* in the standing position did not change substantially for any of the observers or for their mean observations in comparison to the levels of agreement found in the literature. Therefore, the standing position can be regarded as an alternative method to using *My Jump* in adult athletes with added benefits for coaches and practitioners, such as comfort and ease of observation, particularly for massive measurements.

## Figures and Tables

**Table 1:** Between-, within-observer, and method reliability of jump heights derived with *My Jump* 2 for two observation heights.

	Between-Observer Reliability for *h*_1_			Between-Observer Reliability for *h*_2_			Within-Observer, Between-Method, Reliability			Method Reliability
	obs1 vs. 2	obs1 vs. 3	obs2 vs. 3	obs1 vs. 2	obs1 vs. 3	obs2 vs. 3	obs1	obs2	obs3	<*h*_1_> vs. <*h*_2_>
Paired diff. (cm)	0.03	0.11 *	0.08	0.04	0.16 *	0.12	0.31 *	0.31 *	0.35 *	0.32 *
CI-95% lower	−0.08	0.02	−0.07	−0.09	0.05	−0.05	0.22	0.14	0.19	0.22
CI-95% upper	0.14	0.20	0.22	0.17	0.26	0.29	0.39	0.49	0.51	0.42
Paired ES (*g*)	0.01	0.02	0.01	0.01	0.03	0.02	0.05	0.05	0.06	0.05
CI-95% lower	−0.01	0.00	0.00	−0.01	0.01	0.01	0.04	0.04	0.04	0.04
CI-95% upper	0.02	0.02	0.03	0.03	0.02	0.04	0.07	0.07	0.07	0.07
ICC	0.992	0.994	0.985	0.988	0.993	0.980	0.995	0.979	0.983	0.993
CI-95% lower	0.989	0.992	0.981	0.984	0.990	0.974	0.993	0.972	0.977	0.991
CI-95% upper	0.994	0.996	0.989	0.991	0.995	0.985	0.996	0.984	0.987	0.995
CCC	0.992	0.994	0.985	0.988	0.992	0.980	0.993	0.977	0.981	0.992
CI-95% lower	0.989	0.992	0.981	0.984	0.990	0.973	0.991	0.970	0.975	0.989
CI-95% upper	0.994	0.995	0.988	0.991	0.994	0.985	0.995	0.983	0.986	0.998
SEM (cm)	0.55	0.46	0.73	0.67	0.52	0.86	0.44	0.89	0.81	0.51
CI-95% lower	0.50	0.42	0.67	0.61	0.47	0.79	0.40	0.81	0.73	0.46
CI-95% upper	0.61	0.51	0.81	0.75	0.58	0.96	0.46	0.98	0.89	0.56
SEM (stdzed)	0.09	0.08	0.12	0.11	0.09	0.14	0.07	0.15	0.13	0.08
CI-95% lower	0.08	0.07	0.11	0.10	0.08	0.13	0.07	0.13	0.12	0.08
CI-95% upper	0.10	0.08	0.14	0.12	0.09	0.16	0.08	0.16	0.15	0.09
CV (%)	0.97	0.76	1.30	1.16	0.93	1.58	0.85	1.69	1.52	1.05
CI-95% lower	0.85	0.66	1.15	1.01	0.82	1.41	0.75	1.51	1.35	0.94
CI-95% upper	1.09	0.86	1.46	1.31	1.04	1.76	0.95	1.88	1.69	1.15
SDC (cm)	1.51	1.27	2.03	1.87	1.44	2.40	1.21	2.45	2.23	1.41
CI-95% lower	1.38	1.15	1.85	1.70	1.31	2.18	1.10	2.23	2.03	1.28
CI-95% upper	1.68	1.41	2.26	2.07	1.60	2.66	1.34	2.48	2.48	1.56
SWC (cm)	1.20	1.20	1.20	1.21	1.21	1.21	1.20	1.20	1.21	1.20
CI-95% lower	1.07	1.07	1.07	1.08	1.08	1.08	1.08	1.07	1.08	1.08
CI-95% upper	1.31	1.31	1.31	1.33	1.33	1.33	1.32	1.32	1.32	1.32
SNR	2.19	2.62	1.64	1.80	2.34	1.40	2.76	1.33	1.47	2.37

Obs: observer, <*h*>: mean observations from the three observers at observation height *h*, CI-95%: confidence intervals at 95%, ES: effect size, ICC: intraclass correlation coefficient, CCC: concordance coefficient correlation, CV: coefficient of variation, SWC: smallest worthwhile change, SDC: smallest detectable change, SNR: signal to noise ratio, * *p* < 0.05 in the paired differences.

## Data Availability

The data presented in this study are available on reasonable request from the corresponding author.
